# Increased Prevalence of *Methanosphaera stadtmanae* in Inflammatory Bowel Diseases

**DOI:** 10.1371/journal.pone.0087734

**Published:** 2014-02-03

**Authors:** Pascale Blais Lecours, David Marsolais, Yvon Cormier, Marie Berberi, Chantal Haché, Raymond Bourdages, Caroline Duchaine

**Affiliations:** 1 Centre de recherche de l'Institut Universitaire de Cardiologie et de Pneumologie de Québec, Québec, Québec, Canada; 2 Département de biochimie, de microbiologie et de bioinformatique, Faculté des sciences et de génie, Université Laval, Québec, Québec, Canada; 3 Département de médecine, Faculté de médecine, Université Laval, Québec, QC, Canada; 4 CSSS Alphonse-Desjardins, Lévis, Québec, Canada; University of Ulm, Germany

## Abstract

**Background:**

The gut microbiota is associated with the modulation of mucosal immunity and the etiology of inflammatory bowel diseases (IBD). Previous studies focused on the impact of bacterial species on IBD but seldom suspected archaea, which can be a major constituent of intestinal microbiota, to be implicated in the diseases. Recent evidence supports that two main archaeal species found in the digestive system of humans, *Methanobrevibacter smithii* (MBS) and *Methanosphaera stadtmanae* (MSS) can have differential immunogenic properties in lungs of mice; with MSS but not MBS being a strong inducer of the inflammatory response. We thus aimed at documenting the immunogenic potential of MBS and MSS in humans and to explore their association with IBD.

**Methods:**

To validate the immunogenicity of MBS and MSS in humans, peripheral blood mononuclear cells from healthy subjects were stimulated with these two microorganisms and the production of inflammatory cytokine TNF was measured by ELISA. To verify MBS and MSS prevalence in IBD, stool samples from 29 healthy control subjects and 29 patients suffering from IBD were collected for DNA extraction. Plasma was also collected from these subjects to measure antigen-specific IgGs by ELISA. Quantitative PCR was used for bacteria, methanogens, MBS and MSS quantification.

**Results:**

Mononuclear cells stimulated with MSS produced higher concentrations of TNF (39.5 ng/ml) compared to MBS stimulation (9.1 ng/ml). Bacterial concentrations and frequency of MBS-containing stools were similar in both groups. However, the number of stool samples positive for the inflammatory archaea MSS was higher in patients than in controls (47% vs 20%). Importantly, only IBD patients developed a significant anti-MSS IgG response.

**Conclusion:**

The prevalence of MSS is increased in IBD patients and is associated with an antigen-specific IgG response.

## Introduction

The worldwide incidence and prevalence of inflammatory bowel diseases (IBD) is in constant rise [1], [2]. Between 20 000 and 100 000 people are diagnosed with these diseases annually in North America [3]. IBD include two major forms, namely Crohn's disease (CD) and ulcerative colitis (UC). They are characterized by an uncontrolled inflammation of the intestinal mucosa that usually affects the colon (UC) or the total gastrointestinal tract (CD) [4], [5].

The etiology of IBD is not clear, but the role of the gut microbiota is possibly a determining factor of the diseases. The study of intestinal microbiota and its impact on human health has seen an upsurge in interest over the last years. Indeed, evidence suggests that an interplay between microbiota from the gut and the immune system is implicated in IBD [5], [6], [7], [8]. Moreover, there is growing evidence that dysbiosis, i.e an imbalance between commensal beneficial bacteria and pathogens in the gut lumen, is associated with IBD [9]. Indeed, biodiversity of intestinal bacteria from IBD patients is lower than for healthy subjects [10], [11], and some studies demonstrated that the Firmicutes/Bacteroidetes ratio, the two dominant phyla in human gut, is lower in IBD patients compared to healthy subjects [12]. Several bacterial species have been associated with IBD, including species from the Proteobacteria [9] such as adherent-invasive *Escherichia coli* [13], [14], [15], *Campylobacter concisus* [16], [17] and enterohepatic *Helicobacter* [18], [19]. As yet, however, there is no definitive proof for any specific etiological agent in IBD.

An archaeal group, the methanogens, can be found in the digestive system of various mammals, including some humans [20], [21], [22], [23]. Methanogens play an important role in digestion, improving efficiency of polysaccharide fermentation by helping preventing accumulation of acids, reaction end products and gaseous hydrogen [23], [24], [25], [26]. *Methanobrevibacter smithii* (MBS), the dominant archaeal species of the human gut, can constitute up to 11.5% of the total intestinal microbiota [27], but Pochart *et al*. [28] showed that these microorganisms were mostly found in distal colon, an intestinal segment responsible for nonabsorbed material elimination. Despite the significant concentrations of methanogens in some human guts, only one study addressed the prevalence of these microorganisms in IBD [29]. Scanlan *et al*. showed reduced incidence of methanogens in DNA faecal extracts from IBD patients compared to controls, but did not observe any difference in methanogen biodiversity in these two groups.

Dridi *et al*. [22] showed that the methanogens found in human gut microbiota are mainly constituted of MBS, and that *Methanosphaera stadtmanae* (MSS) was present in only a small fraction of these samples. We recently demonstrated that these two methanogen species have a differential immunogenic potential in lungs of mice [30]. Indeed, while MSS can sustain a myeloid dendritic cell response associated with a strong granulocytic response, MBS triggers a lymphocytic response devoid of inflammatory cells. It was also shown that methanogens and their components could modulate immune responses in the human oral cavity [31], [32]. It is thus tempting to speculate that archaeal species could be involved in the immunological homeostasis of the gut.

Given that MBS and MSS can be found in human gut and that they can differently modulate the immune system in the lungs of mice, our aims were to first confirm immunogenicity of MBS and MSS humans, and secondly, to compare the presence of these two microorganisms in stool samples of patients and control subjects. Quantification of antigen-specific antibodies in the plasma of subjects was also performed to associate the MBS and MSS composition of the stool samples with the peripheral blood immune response. We show that both MBS and MSS can induce the release of the proinflammatory cytokine TNF in peripheral blood mononuclear cells (PBMC), with MSS inducing a 4-fold stronger response than MBS. We also show an increased prevalence of MSS-positive stool samples in IBD patients compared to controls subjects, which was not the case for total methanogens and MBS. Plasma from patients positive for MSS in stool samples contained also higher levels of antigen-specific IgGs compared to control subjects.

## Materials and Methods

### Volunteers recruitment

Thirty patients diagnosed with inflammatory bowel disease were recruited by the gastroenterology service of CSSS Alphonse-Desjardins Hospital and 30 healthy control subjects without any history of bowel disease were recruited by the CRIUCPQ research team. From these healthy controls, eleven were randomly chosen for a first series of experiments aiming to validate the immunogenicity of MBS and MSS in humans. Exclusion criteria for volunteers included a body mass index over 30 [33] and an antibiotic treatment within one month before the sampling since these two factors can influence microbiota. Control subjects were paired with patients for age (±5 years) and gender. Data obtained from recruited patients included date of IBD diagnostic, medication, type and status of the inflammatory bowel disease and surgical history.

### Ex vivo stimulation of PBMCs with MBS and MSS

Blood from eleven healthy subjects (randomly chosen from the 30 healthy controls recruited for the main project) was sampled and Lymphocyte Separation Medium gradient (Wisent Bioproducts, St-Bruno, Canada) was performed for PBMC isolation. PBMC (12×10^6^ cells/ml) were cultivated at 37°C in a 5% CO_2_ atmosphere in RPMI-1640 medium (Wisent Bioproducts, St-Bruno, Canada) supplemented with 10% heat-inactivated fetal bovine serum and 1% penicillin-streptomycin. Cells from every subject were either not stimulated or stimulated for four hours with 0.05 mg/ml of saline-resuspended lyophilized MBS or MSS. After stimulation, cell-free supernatants were collected and stored at −80°C. To compare immunogenicity of MBS and MSS, the inflammatory cytokine TNF was measured in cell supernatants using Human TNF-alpha DuoSet ELISA Kit (R and D systems, Minneapolis, MN).

### Sampling and processing methods

Stool samples of approximately 40 g were obtained from patients and control subjects. Samples were kept on ice, shipped by mail and received within 48 hours. A small fraction of the stool sample was dried to calculate dry mass. The samples were homogenized in a sterile stomacher Filtra-Bag (Labplas, Quebec, Canada) for 30 s in a Stomacher Mix 1 (Aes Laboratoire, Bruz, France) with trice the volume of phosphate buffered saline containing 0.05% Tween 20. The liquid homogenate was aliquoted in 0.5 ml fractions, centrifuged at 5000 g for 10 min and pellets were kept frozen at −20°C until DNA extraction. Plasma was also recovered from blood samples of 18 healthy control subjects (9 bearing intestinal MBS and 4 bearing intestinal MSS) and 17 patients (6 bearing intestinal MBS and 9 bearing intestinal MSS). Subjects were divided into four groups for analysis, i.e controls negative for MBS or MSS in stool samples, controls positive for MBS or MSS in stool samples, patients negative for MBS or MSS in stool samples and patients positive for MBS or MSS in stool samples. Indirect ELISA was used to measure MBS- and MSS-specific IgGs as previously described [30] in three plasma dilutions (10, 100 and 500X). Specific IgGs were quantified in each plasma dilution with optical density (OD). Each plate contained blank and internal controls. Total IgGs were also quantified using Human IgG total Ready-SET-Go! ELISA kit (Affymetrix eBioscience, San Diego, CA).

### Total DNA extraction

Powerlyzer PowerSoil DNA extraction kit (MO BIO, Carlsbad, CA), which features a high efficiency inhibitor removal technology, was used for stool total DNA extraction according to the manufacturer. Heating step at 65°C for 10 min was added before the bead beating step, which was performed in a MixerMill MM300 (Retsch, Haan, Germany) at speed 20 for 20 min. DNA extracts were conserved at −20°C until further use.

### Quantitative real-time PCR

Quantitative real-time PCR was performed on a DNA Engine CFX (Bio-Rad Laboratories, Mississauga, Canada). All PCR reactions were conducted using the iQSupermix (Bio-Rad Laboratories, Hercules, CA). All DNA samples were diluted 1/10 before performing the assay, to avoid PCR inhibition. Cycle thresholds were determined automatically by the software.

Primers specific for the mtaB1 gene (coenzyme M methyltransferase) of MSS [34] were designed with Primer Premier software (Premier Biosoft, Palo Alto, CA). The mtaB gene has only been described in *Methanosarcina* organisms and MSS [34]. Because the mtaB gene of *Methanosarcina* gender is very similar to mtaB1, the two sequences of the genes were compared with Bioedit which demonstrated significant differences between the two genes. Primers were also tested by PCR on MSS, MBS as well as several *Methanosarcina* species (*M. acetivorans*, *M. mazei* and *M. thermophila*), and were specific only to MSS. Amplicons were sequenced to validate the specificity of the primers and the protocol. Therefore, mtaB1 was determined to be a good target gene for MSS detection. A total of 12.5 µl of iQ SYBR green Supermix (Bio-Rad Laboratories, Hercules, CA) and 5 µl of the DNA template were used in a 20 µl reaction mixture. The amplification program used for this PCR was as follow: one hold at 95°C for 3 min and then 40 cycles at 95°C for 30 s, 55.5°C for 30 s, fluorescence acquisition, and 72°C for 45 s. A melting curve program was also performed to verify amplicon specificity using the following program: 40°C to 95°C, read every 0.2 s, hold 1 s. Samples were considered positive for mtaB1 genes when the melting temperature was around 81°C. A standard curve was processed using ten-fold genomic DNA dilutions of MSS cultured in our laboratory.

PCR specific for MBS was conducted using the primers, probe (Table 1) and amplification program published by Johnston *et al*. [35]. A standard curve was processed using ten-fold genomic DNA dilutions of MBS cultured in our laboratory.

**Table 1:** Primers and probes used in the study.

Gene target	Primers and probes	Nucleotide sequence (5′-3′)	Reference
Bacterial 16S rRNA	EUB F	GGT AGT CYA YGC MST AAA CG	Bach *et al*., 2001
	EUB R	GAC ARC CAT GCA SCA CCT G	Bach *et al*., 2001
	Probe EUB	FAM-TKC GCG TTG CDT CGA ATT AAW CCA C-TAMRA	Bach *et al*., 2001
MtaB1 (*M. stadtmanae*)	MSS 122F	CTA ACA TCA AAG TAG CTC C	This study
	MSS 414R	TCC TCT AAG ACC GTT T	This study
NifH (*M. smithii*)	Mnif 202 F	GAA AGC GGA GGT CCT GAA	Johnson *et al*., 2010
	Mnif 353R	ACT GAA AAA CCT CCG CAA AC	Johnson *et al*., 2010
	Mnif Probe	[FAM]- CCG GAC GTG GTG TAA CAG TAG CTA –[BHQ]-1	Johnson *et al*., 2010
McrA (Methanogens)	mlas	GGT GGT GTM GGD TTC ACM CAR TA	Steinberg et al. 2008
	mcrA-rev	CGT TCA TBG CGT AGT TVG GRT AGT	Steinberg et al. 2008

Total methanogens were quantified using primers (Table 1) and modified qPCR protocol from Steinberg *et al*. [36], [37]. The amplification program used was as follow: one hold at 95°C for 3 min and then 40 cycles at 95°C for 30 s, 59°C for 30 s, fluorescence acquisition, and 72°C for 30 s. The melting curve was performed as mentioned above, with a melting temperature specific around 82°C.

To quantify total bacteria, PCR was conducted using primers and probe (Table 1) from Bach *et al*. [38]. Amplification programs were performed as mentioned above, with annealing temperature of 62°C. A standard curve was processed using ten-fold serial DNA dilutions of plasmid containing *Escherichia coli* (ATCC 25922) 16S rRNA gene sequences.

### Statistical analysis

Bacterial data are expressed using median with interquartile range. The analyses of categorical variables were performed using the Fisher's exact test. Bacterial counts were analysed using a Student's t-test after a log transformation to stabilize variances. Blood sampling data were analysed using one-way ANOVA. Posteriori comparisons were performed with the Tukey's technique comparisons. The results were considered significant with p-values ≤.05. All analyses were conducted using the statistical package SAS v9.3 (SAS Institute Inc, Cary, NC).

### Ethical considerations

Recruitment and experimental protocols were reviewed and approved by the Ethic Committee of CSSS Alphonse-Desjardins (approval # CER-1213-002). Written consent was obtained from every participant.

## Results

### Subjects' characteristics

After the recruitment of 30 patients and their controls, one pair had to be excluded from the study and analysis, since we afterwards learned that one control had recently suffered from cancer and still had adverse side effects from chemotherapy. The absence of such status was confirmed in the other 29 pairs of volunteers. Eighteen patients suffering from CD (3 male and 15 female) and 11 with UC (7 male and 4 female) were thus included in the study (Table 2). The age of each group of patients was similar: average 42.1±2.7 years for patients with CD vs 41.1±5.3 years for patients with UC. Control subjects were paired for age and sex with the patients. Eight patients (two males and two females suffering from CD and two males and two females from UC) had active disease during the sampling. All patients were under pharmaceutical treatment, which included antimetabolites, anti-TNFα antibodies, corticosteroids, immunosuppressors, analogue of somatostatin and supplement of calcium and folic acid (Table 2). Two females and one male with CD had an intestinal surgical treatment from one month to up to seven years before the sampling.

**Table 2:** Clinical status of recruited patients.

	Crohn's disease (*N* = 18)		Ulcerative colitis (*N* = 11)	
Characteristics	Male	Female	Male	Female
N	3	15	7	4
Age (years ± SEM)	33.0±2.5	42.2±2.7	41.6±8.0	40.3±5.6
Active disease status	2	2	2	2
Therapeutic treatment				
Antimetabolites	1	6	4	3
Anti-TNFα antibodies	2	9	2	1
Corticosteroids	1	2	2	2
Anti-inflammatory	0	1	4	1
Immunosuppressor	1	3	1	0
Analogue of somatostatin	0	1	1	0
Calcium supplement	1	3	1	2
Folic acid	1	0	1	0
Surgical treatment	1	2	0	0

### Validation of immunogenicity of MBS and MSS in humans

**Figure 1:**
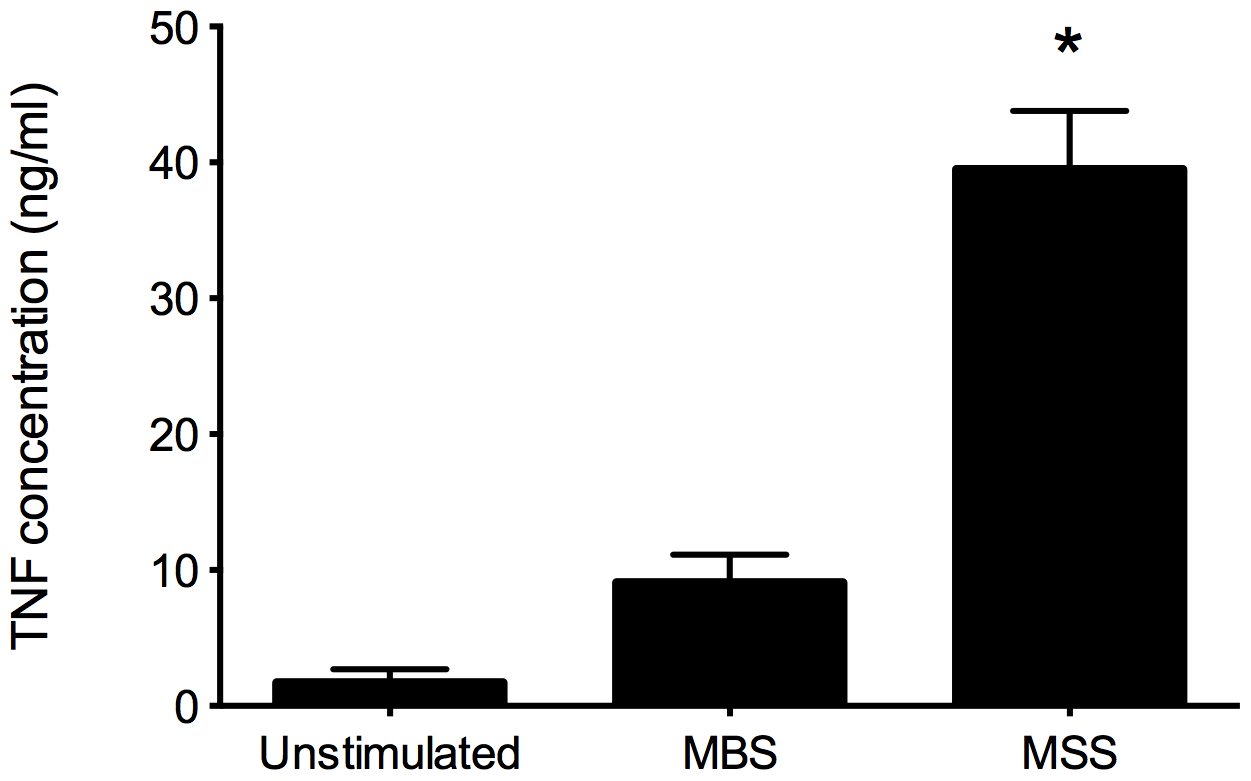
TNF concentrations in supernatants of non-stimulated and MBS- or MSS-stimulated PBMC from healthy human subjects. TNF was quantified by direct ELISA (average ± SEM). Cells from eleven subjects per group were analyzed. p<0.001.

Concentrations of the inflammatory cytokine TNF were measured in supernatants of non-stimulated and MBS- or MSS-stimulated PBMC from healthy human subjects (Figure 1). Very low levels of TNF were detected in supernatant of non-stimulated cells (average of 1.73±0.92 ng/ml). Supernatants of cells stimulated with MSS showed much higher levels of TNF (average of 39.5±4.3 ng/ml) compared to cells stimulated with MBS (average of 9.1±1.9 ng/ml).

### MSS-specific DNA amplification with qPCR method

We developed a new qPCR method to detect and quantify DNA of MSS. A specific PCR protocol for this archaeal species was only described once [29], but in that study the authors were unable to amplify MSS DNA from stool samples. Primers specific for MSS quantification (MSS 122F and MSS 414R) and optimized amplification protocol designed in the current study allowed successful amplification of the mtaB1 gene of this microorganism. This primer set was very specific for MSS (100% homology) when the sequences were compared with MSS sequences available in databases using BLASTN [39] from the National Center of Biotechnology Information (http://www.ncbi.nlm.nih.gov/BLAST/). Primers were also specific for MSS when tested on other methanogenic species (no amplification on MBS, *M. acetivorans*, *M. mazei* and *M. thermophila*; not shown). The standard curve was linear from 1 to 10^6^ gene copies per reaction (efficiency = 96.2%, r^2^ = 0.997). The limit of detection (LOD) for this PCR protocol in stool samples was 10^3^ cells per gram of sample.

### Concentrations of total bacteria in stool samples from patients and controls

Total bacteria (Table S1) were quantified in the stool samples from IBD patients and healthy control subjects and were used as reference values for microbial burden to adjust species concentrations between samples. There was no difference in the total number of bacteria in samples from control subjects compared to patients (medians of 9.3×10^10^ vs 1.9×10^11^ bacterial 16S rRNA genes per gram of dried stool, respectively).

### Concentrations and prevalence of methanogenic species in stool samples of controls and IBD patients

Total methanogens, MBS and MSS were detected (Table 3) and quantified (Table S1) in the stool samples from IBD patients and healthy control subjects. Median concentrations of total methanogens, MBS and MSS-specific genes per gram of dried stool were under the LOD for patients and control subjects. The number of samples positive for MBS, i.e above the LOD at 10^4^ cells per gram of stool, was not different for patients (10 out of 29) compared to control subjects (13 out of 29). However, MSS-positive samples were 3 times more frequent in patients (14 out of 29) than in controls (5 out of 29) (Table 3) (p<0.05).

**Table 3:** Comparison of the presence of MSS and/or MBS in stool samples from control subjects and patients.

	Number of positive samples			
MSS/MBS	−/−	+/+	−/+	+/−
Controls (n = 29)	12	1	12	4
Patients (n = 29)	11	6	4	8

Results are expressed as numbers of positive samples for MSS and/or MBS for each group. Twenty-nine subjects per group were analyzed. P<.05 when patients were compared to controls for prevalence distribution of MBS and MSS.

### Patients' characteristics and archaeal gut microbiota

The association between patient's characteristics and concentrations of microorganisms from their stool samples was evaluated. Age, gender, disease type and status, and drug treatments were considered in the analysis (Table S1). Younger male patients tended to have higher concentrations of MSS than the older subjects. This association can probably be explained by the results of two young subjects with very high MSS levels. Although we observed a tendency of mesalazine-treated patients to have higher concentrations of MSS in stool samples, no significant impact of drug treatment on microorganisms' prevalence and concentrations were found (Table S1).

### Antigen-specific IgGs in plasma of controls and patients

**Figure 2:**
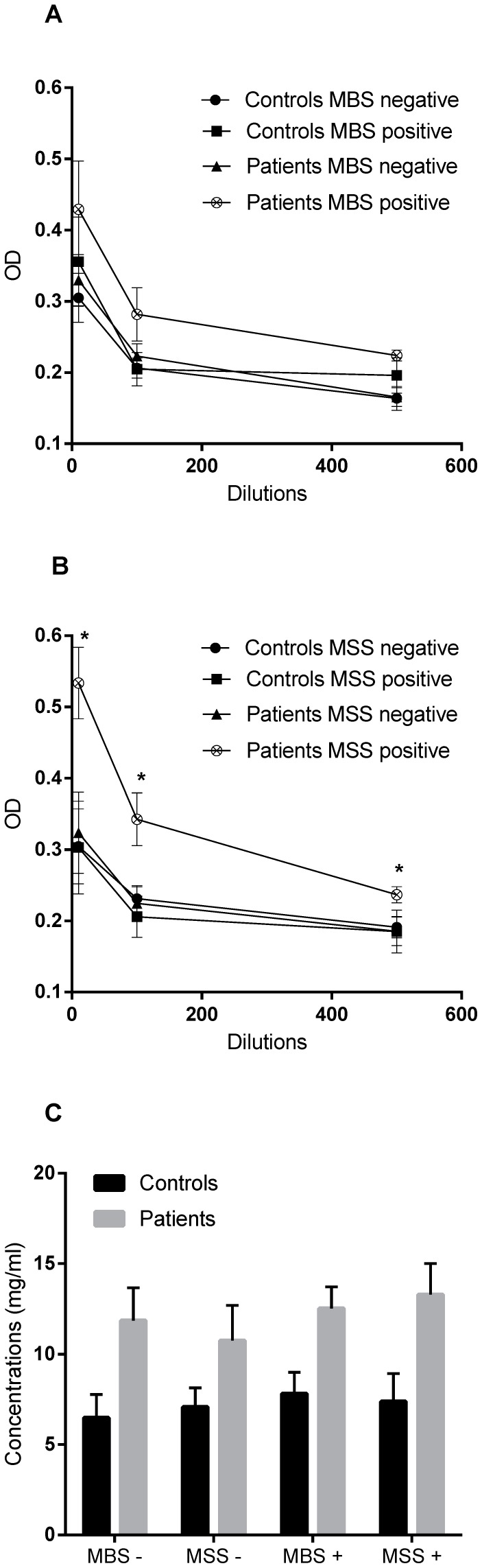
IgG response in plasma of controls subjects and IBD patients. Titers of A) MBS- and B) MSS-specific IgGs. C) Quantification of total IgGs. Results are expressed as average ± SEM. Eighteen controls (9 positive for MBS and 4 positive for MSS in stool samples) and seventeen patients (6 positive for MBS and 9 positive for MSS in stool samples) were analyzed. p<0.05.

Peripheral immune response to MBS and MSS was evaluated by measuring antigen-specific IgGs in plasma from control subjects and patients (Fig. 2). Similar quantities of MBS-specific IgGs were detected in the plasma of control subjects and patients. IgG levels were not influenced by the presence or absence of this microorganism in their stool samples (Fig. 2A). However, levels of MSS-specific IgGs were higher at every plasma dilution for patients positive for MSS in the stool samples (average OD of 0.533±0.05 for highest dilution) compared to negative patients (average OD of 0.324±0.05 for higher dilution) and control subjects (average OD of 0.304±0.05 for highest dilution) (Fig. 2B) (p<0.05). To control IgG background of control and patients, total IgGs from every subjects were quantified (Fig. 2C). As suspected, plasma from control subjects contained lower levels of total IgGs (average of 7.1±0.87 mg/ml) than that of patients (average of 12.1±1.3 mg/ml). Moreover, patients negative for MBS or MSS in their stool samples had similar total IgGs quantities (averages of 11.9±1.8 and 10.8±1.9 mg/ml, respectively) than those positive for MBS or MSS (averages of 12.5±1.2 and 13.3±1.7 mg/ml). These results suggest that the total IgG levels do not explain immune status of patients and therefore peripheral response is specific to MBS or MSS.

## Discussion

Several microorganisms from the gut have been associated with IBD but their role in these diseases is still unknown. Our team recently showed that methanogenic archaeal species MBS and MSS, which can be major components of the human and animal intestinal microbiota, are present in bioaerosols samples and have immunogenic properties in the lungs of mice. The current report validates these properties on human PBMCs and is the first to demonstrate a differential prevalence of MSS in stool samples of patients suffering from IBD compared to healthy control subjects. The results of this study show that stool samples from patients have similar quantities of total bacteria as control subjects; similar incidence of methanogens and MBS, and higher prevalence of MSS. Moreover, we demonstrate that intestinal-MSS bearing patients develop higher levels of plasma specific IgGs compared to intestinal-MSS bearing healthy control subjects.

Methanogens are a significant part of the human microbiota in some individuals and have been associated with diseases, including periodontitis (*Methanobrevibacter oralis*) [31], or negatively associated to others, such as obesity (*Methanobrevibacter smithii*) [33]. However, these microorganisms were never associated with any intestinal disease. Previous studies aimed to demonstrate a possible role of methanogens in IBD [29], but the potential impact of an imbalance between MSS and MBS alone was never observed. Indeed, Scanlan *et al*. [29] demonstrated that a lower proportion of patients were positive for methanogens in their gut compared to controls. Our results confirm that observation. Our data show that this decrease is at the expense of the usually much higher numbers of MBS, which tended to be decreased in IBD. Unlike the study from Scanlan *et al*. [29], we were able to amplify and quantify MSS DNA from stool DNA extracts, suggesting higher sensitivity for the primers we designed. Moreover, our data suggest that PCR protocols used for MBS and MSS detection are more efficient than the PCR protocol used for total methanogen quantification since some samples were positive for MBS or MSS primers but negative with the total methanogen primers (table 3).

When looking at methanogen-specific species we found an association between MSS and IBD, which would have been missed had we only described total methanogens. As reviewed by numerous studies, commensal bacteria could be a source of antigens driving the immune response in IBD [10], [40], [41]. Studies are needed to verify if MSS antigens could also initiate or maintain the inflammation in IBD. This hypothesis was based on our previous observation in the lungs of mice [30] and results obtained in this study suggest that MSS can have a significantly stronger immunogenic potential than MBS. Indeed, we demonstrated that MSS induces inflammation in the lungs of mice while MBS does not [30]. This hypothesis is strengthened by the current finding that MSS but not MBS is highly proinflammatory in human peripheral blood mononuclear cells; and that a strong IgG response to MSS, not MBS, is seen in IBD patients compared to controls. Given that several associations of seroreactivity tests against specific antigens have been described in IBD [42], [43], [44], it is tempting to speculate that MSS could be involved in the pathogenic sequence of IBD in a subset of patients. The observed difference could also be a consequence of the disease rather than a cause.

Drug treatments influence methanogenic microbiota from the gut of IBD patients. Swidsinski *et al.* [45] demonstrated that mesalazine-treated IBD colitis patients had fewer mucosal bacteria than non-treated subjects and controls. They also showed that azathioprine, an immunosuppressor, is associated with a 3-log increase of intestinal mucosal bacteria compared to controls. Although we observed a tendency of mesalazine-treated patients to have higher concentration of MSS, drug treatment for IBD did not seem to impact on methanogen prevalence and concentration in the gut of patients in this study. No difference was observed between active disease and remission status. However, the low number of active disease patients makes it difficult to conclude on the impact of the disease status.

Gender distribution of recruited patients are representative of IBD prevalence in the general population and data acquired are thus valid [3]. Limits in DNA extraction approaches and PCR sensitivity in rich microbial environments where inhibitors are present led to a limit of detection (LOD) similar to what we usually observed [46], [47], [48]. Our approach included a larger sample homogenization rather than a sub-sampling for molecular analyses, that could have increased the representativeness of the results and reduced the impact of sub-sample variability. Finally, we report in this article an effective qPCR protocol for MSS-specific DNA amplification. The qPCR protocols used for specific detection of MBS and MSS seemed to be more sensitive than qPCR protocol used for total methanogens amplification. Indeed, more samples were negative for total methanogens than for MBS or MSS. This could also be explained by the different limits of detection.

## Conclusion

We demonstrate for the first time that MSS, a methanogen species from the human gut, is distributed differentially in healthy individuals compared to patients suffering from IBD. We also confirmed the immunogenic potential of MSS in human PBMCs and a strong seroreactivity to MSS in IBD patients. Considering the pro-inflammatory properties of this archaea, we believe that the nature of its interplay with IBD should be further investigated.

## Supporting Information

Table S1 **Complete results for all individual subjects.**
(DOCX)
