# Supplementary material for: Increased Prevalence of Methanosphaera stadtmanae in Inflammatory Bowel Diseases
Source: PLoS One. 2014 Feb 3;9(2):e87734. doi: 10.1371/journal.pone.0087734 (PMC3912014; doi:10.1371/journal.pone.0087734)
Supplement: Table S1 — Complete results for all individual subjects. (DOCX) [file pone.0087734.s001.docx]

**Table S1.** Complete results for all individual subjects

|  |  |  |  | Microorganisms/g dried stool | | | |
| --- | --- | --- | --- | --- | --- | --- | --- |
|  |  | Sexe | Age | Bacteria | Methanogens | MBS | MSS |
| Patients | 1^*,§,Ŧ, Ψ^ | M | 31 | 4.23E+11 | 1.14E+06 | <LOD | 2.55E+04 |
|  | 2^§^ | M | 30 | 2.60E+10 | <LOD | <LOD | 8.27E+03 |
|  | 3^*, §^ | M | 23 | 9.43E+11 | <LOD | <LOD | 6.92E+04 |
|  | 4^*, Ŧ, ε, Ψ^ | F | 38 | 2.53E+10 | 3.46E+08 | 9.61E+07 | <LOD |
|  | 5^Ŧ, ε, Φ, Ψ^ | M | 54 | 1.95E+11 | 7.33E+05 | <LOD | <LOD |
|  | 6^§^ | F | 58 | 8.23E+11 | <LOD | <LOD | <LOD |
|  | 7^*,§,Ŧ,Ψ^ | F | 38 | 4.83E+10 | 6.98E+05 | 5.77E+04 | 4.51E+03 |
|  | 8^*, Ŧ, Ψ^ | F | 28 | 9.00E+10 | <LOD | <LOD | <LOD |
|  | 9^*,§,ε^ | M | 18 | 2.88E+11 | 4.97E+05 | <LOD | 3.47E+04 |
|  | 10^ε^ | F | 40 | 2.59E+11 | <LOD | <LOD | <LOD |
|  | 11^*,§^ | F | 43 | 5.76E+11 | <LOD | <LOD | 1.83E+04 |
|  | 12^*^ | F | 38 | 1.87E+11 | <LOD | <LOD | <LOD |
|  | 13^*,§^ | F | 53 | 2.06E+11 | <LOD | <LOD | <LOD |
|  | 14^§^ | F | 47 | 1.70E+11 | <LOD | <LOD | <LOD |
|  | 15^Φ, Ψ^ | F | 51 | 1.91E+11 | 1.02E+06 | 6.07E+04 | <LOD |
|  | 16^Φ, Ψ^ | F | 45 | 1.99E+11 | 3.62E+05 | 2.31E+06 | 2.98E+04 |
|  | 17^ε^ | M | 72 | 2.54E+10 | 1.54E+08 | 2.76E+07 | 9.43E+06 |
|  | 18^§^ | F | 26 | 2.48E+11 | <LOD | <LOD | <LOD |
|  | 19^§, ε^ | F | 35 | 2.01E+11 | <LOD | <LOD | <LOD |
|  | 20^*,§^ | F | 27 | 1.42E+12 | <LOD | <LOD | 8.34E+04 |
|  | 21^*,Ŧ^ | F | 24 | 1.41E+10 | <LOD | <LOD | <LOD |
|  | 22^*^ | M | 20 | 5.70E+10 | 4.61E+05 | 4.92E+05 | <LOD |
|  | 23^*, Δ^ | M | 48 | 1.95E+12 | 3.88E+05 | 2.56E+05 | <LOD |
|  | 24^ε^ | M | 56 | 8.40E+09 | 1.72E+08 | 1.57E+08 | 1.58E+07 |
|  | 25^§, Δ^ | F | 49 | 1.29E+11 | <LOD | <LOD | 4.33E+04 |
|  | 26^Φ^ | M | 38 | 8.89E+10 | <LOD | <LOD | <LOD |
|  | 27^*^ | F | 55 | 3.99E+10 | 3.36E+08 | 2.68E+08 | 1.67E+04 |
|  | 28^ε^ | F | 49 | 9.76E+10 | <LOD | 6.68E+04 | 1.84E+04 |
|  | 29^§^ | F | 50 | 3.83E+10 | <LOD | <LOD | 5.83E+03 |
| Controls | 1 | M | 29 | 1.41E+10 | <LOD | <LOD | <LOD |
|  | 2 | M | 28 | 1.48E+11 | <LOD | <LOD | 1.45E+04 |
|  | 3 | M | 26 | 3.62E+11 | 9.05E+08 | 5.13E+08 | <LOD |
|  | 4 | F | 35 | 4.38E+10 | 1.34E+08 | 5.43E+07 | <LOD |
|  | 5 | M | 60 | 8.87E+11 | 4.69E+08 | 3.72E+05 | <LOD |
|  | 6 | F | 57 | 1.01E+10 | 2.17E+08 | 1.29E+08 | <LOD |
|  | 7 | F | 35 | 5.34E+10 | 8.14E+08 | 3.62E+08 | <LOD |
|  | 8 | F | 28 | 4.03E+11 | 9.25E+04 | <LOD | 2.10E+04 |
|  | 9 | M | 24 | 2.62E+11 | <LOD | <LOD | <LOD |
|  | 10 | F | 38 | 2.83E+10 | <LOD | <LOD | <LOD |
|  | 11 | F | 41 | 3.22E+11 | <LOD | <LOD | <LOD |
|  | 12 | F | 34 | 9.07E+10 | <LOD | <LOD | <LOD |
|  | 13 | F | 55 | 5.94E+10 | 2.65E+08 | 1.72E+09 | <LOD |
|  | 14 | F | 45 | 9.28E+10 | 1.15E+09 | 3.98E+08 | <LOD |
|  | 15 | F | 53 | 1.56E+11 | <LOD | <LOD | <LOD |
|  | 16 | F | 45 | 3.34E+10 | 6.29E+08 | 1.75E+08 | <LOD |
|  | 17 | M | 72 | 1.27E+11 | 3.31E+05 | <LOD | <LOD |
|  | 18 | F | 24 | 1.54E+11 | <LOD | <LOD | <LOD |
|  | 19 | F | 32 | 6.59E+10 | <LOD | <LOD | <LOD |
|  | 20 | F | 27 | 2.04E+11 | <LOD | <LOD | 1.45E+04 |
|  | 21 | F | 23 | 1.07E+11 | <LOD | <LOD | <LOD |
|  | 22 | M | 18 | 1.96E+10 | 5.96E+06 | 6.45E+06 | <LOD |
|  | 23 | M | 48 | 6.54E+11 | 3.71E+07 | 2.21E+07 | 1.06E+04 |
|  | 24 | M | 56 | 6.25E+10 | 1.02E+09 | 1.07E+09 | <LOD |
|  | 25 | F | 49 | 7.83E+10 | <LOD | <LOD | 1.05E+04 |
|  | 26 | M | 38 | 5.92E+10 | 6.20E+08 | 4.62E+08 | <LOD |
|  | 27 | F | 55 | 1.35E+11 | <LOD | <LOD | <LOD |
|  | 28 | F | 50 | 1.94E+11 | 2.26E+05 | <LOD | <LOD |
|  | 29 | F | 51 | 3.74E+10 | 5.61E+08 | 3.11E+08 | <LOD |

Symbols represent treatments received by patients. *: antimetabolites, §: anti-TNFα antibodies, Ŧ: corticosteroids, Ψ: calcium supplement, ε: anti-inflammatory, Φ: immunosuppressor, Δ: A

analogue of somatostatin
